# Development and Validation of an Indian Nutrition and Food Literacy Tool (INFOLIT) for adolescents

**DOI:** 10.3389/fnut.2025.1626673

**Published:** 2025-11-13

**Authors:** Simran Yadav, Archana Konapur, Thirupathi Reddy Mokalla, SubbaRao M. Gavaravarapu

**Affiliations:** Nutrition Information, Communication and Health Education (NICHE) Division, Indian Council of Medical Research-National Institute of Nutrition, Hyderabad, Telangana, India

**Keywords:** nutrition literacy, food literacy, health literacy, adolescents, tool development, content validity

## Abstract

**Background:**

Nutrition and food literacy (NFL) are interconnected yet distinct concepts concerning the capability to comprehend and utilize knowledge pertaining to nutrition and food. NFL is a significant driver that shapes an individuals’ diet. This is more so among adolescents whose dietary choices leave a lasting impact on nutritional and health outcomes in adulthood. Assessing NFL can help design tailored interventions to empower adolescents to make informed dietary decision. However, there is lack of standardized tools to assess NFL among adolescents, especially in India.

**Objective:**

To develop and validate an Indian Nutrition and Food Literacy Tool (INFOLIT) for adolescents with a focus on key literacy domains.

**Materials and methods:**

This five-phased cross-sectional sequential exploratory study was conducted among adolescents (13–15y) in Hyderabad, Telangana. In Phase-1, an item-pool was created for tool development through literature review and it was tested for content validity in phase-2 by experts (*n* = 5) using a Content Validity Index (CVI) and Cognitive Interviews (CIs) with adolescents (*n* = 15). In phase-3, Psychometric tests (Cronbach’s *α*, item and factor analysis) were performed among 400 adolescents. In phase-4, reliability was assessed (*n* = 30) using the Intra-class Correlation Coefficient (ICC) and Bland–Altman plot was tested and in Phase-5, the tool was graded using tertile-based stratification.

**Results:**

The initial item pool included 98 items into two domains - Cognitive and Skill-based for creating framework. The cognitive-domain had one dimension- Knowledge; whereas, the skill-domain had three dimensions-(i)functional, (ii)interactive and (iii)critical. Content validity and cognitive interviews eliminated 13 items. The item difficulty (p) and item discrimination (D) indices ranged 0.10–0.98 and 0.00–0.88, respectively, with strong internal consistency (Cronbach’s *α* = 0.81). Due to sample size limitations, factor analysis was used only for item reduction. These steps further reduced 12 items, resulting in a 73-item pool. The ICC was 0.895, and Bland–Altman plots revealed negligible mean biases indicating good reliability. The scores were categorized as low (≤40), medium (41–55), and high (≥56) out of 73.

**Conclusion:**

The INFOLIT is a validated and culturally relevant tool for assessing adolescent NFL and can be adapted for use in various Indian regions and contexts after minor modifications.

## Introduction

Adolescence is a transformative phase marked by rapid physical, psychological, and social development, with lasting consequences for an individual’s health across the life course. Today’s adolescents face a complex nutritional landscape characterized by the coexistence of undernutrition, overnutrition, and micronutrient deficiencies, challenges largely shaped by their food environment. Increasing exposure to foods high in salt, fat, and sugar (HFSS), driven by taste, affordability, accessibility, and aggressive marketing strategies, is one the major factors contributing to the surge in consumption of ultra-processed foods (UPFs) among this demographic (1). Between 2011 and 2021, the UPF sector in India grew at a Compound Annual Growth Rate (CAGR) of 13.37%, fueled by celebrity-led, emotion-based marketing that portrays HFSS products as aspirational, cool, and fun, influencing dietary behaviors and normalizing eating away from home (2, 3). These dietary patterns, shaped usually early in life, significantly contribute to over-weight, obesity, Non-Communicable Diseases (NCDs) during adulthood (4). National surveys reflect the health toll of these patterns: the Comprehensive National Nutrition Survey (CNNS) (2019) reported that 4.9% of Indian adolescents are overweight, 10.4% have Pre-Diabetes and 4.9% have Pre-Hypertension, highlighting the urgent need for preventive strategies.

Adolescents’ food choices are shaped not only by peers, family environment, and schools, but also by an individual’s Nutrition Literacy (NL) and Food Literacy (FL) (5). NL refers to the ability to understand nutrition information, whereas FL extends this to include skills and confidence to effectively act on such knowledge (6–8). NL can thus be viewed as a subset of FL (9). Together, these literacies equip adolescents to navigate complex food environments, resist unhealthy marketing, and make autonomous, healthier choices.

Building NFL from the preschool years can help establish lifelong healthy habits and schools, where adolescents spend much of their time, offer a prime setting to develop NFL (5). Yet in India, food and nutrition education are neither integrated nor prioritized in schools (10). Therefore, adolescents lack the cognitive skills needed to critically evaluate marketing claims or interpret food labels, leaving them vulnerable to aggressive FMCG (fast-moving consumer goods) marketing (10, 11). Evidence underscores this gap: A school-based survey across tier-1 cities found that only one-third of adolescents reported reading nutrition labels while selecting packaged foods and a 2025 study showed that fewer than 15% of adolescent girls demonstrated adequate knowledge of balanced diets (12, 13). These findings point to a concerning gap in basic nutrition knowledge, skills, and application of the same on the food choices, among Indian adolescents, highlighting the urgent need for validated tools and early interventions in schools.

Most of the existing tools tend to focus on either NL or FL, with only a few addressing both dimensions comprehensively (6–8). Globally, only a limited number of validated and reliable tools exist to assess NL and FL in children and adolescents, including the Modified Food and Nutrition Literacy Tool (M-FNLIT) in Iran, the Food and Nutrition Literacy Questionnaire for Chinese School-age Children (FNLQ-SC), the Thai Nutritional Literacy Assessment Tool for Adolescents (Thai-NLAT), the Preschool Food Literacy Assessment Tool (Preschool-FLAT), the Menu Board Literacy Instrument, and the Food Label Literacy for Applied Nutrition Knowledge (FFLANK) (7, 14–18). However, most existing instruments, particularly those developed in low- and middle-income countries, are highly context-specific, reflecting the dietary practices, cultural norms of the countries in which they were developed. For example, items related to processed meat consumption like sausages or traffic light labelling systems based on dietary guidelines are not relevant to the Indian context, where such foods and frameworks are not similar (7, 19).

This highlights a key limitation: tools developed in other high-income and LMICs may not be directly applicable in the Indian context, as they do not account for the unique dietary patterns, food availability, and cultural norms around food in India. This absence of culturally appropriate, validated, and reliable NFL tools for Indian adolescents is a critical research gap. Without such a tool, it is difficult to assess literacy levels, design targeted interventions, or equip adolescents with the knowledge and skills needed for healthier eating behaviors. Given the rising burden of nutrition-related health issues and adolescents’ susceptibility to poor dietary choices and aggressive food marketing, developing such an instrument is both just timely and essential. Therefore, this study aims to (i) adapt existing NFL constructs to the Indian context, and (ii) develop and test the reliability and validity of the culturally appropriate tool, to assess NFL among Indian adolescents.

## Materials and methods

### Theoretical framework

This study drew on the Nutbeam model of health literacy, which identifies three levels - Functional, Interactive and Critical (20). Wang et al. (21) further define these as: Functional, “The capacity to access, understand and use nutritional information”; Interactive, “The capacity to take action to obtain and exchange nutrition information through interaction and engaging in various forms of communication”; and Critical, “The capacity to critically evaluate and appraise nutritional information, advice and recommendations from various sources with the right perspective” (21).

**Figure 1:**
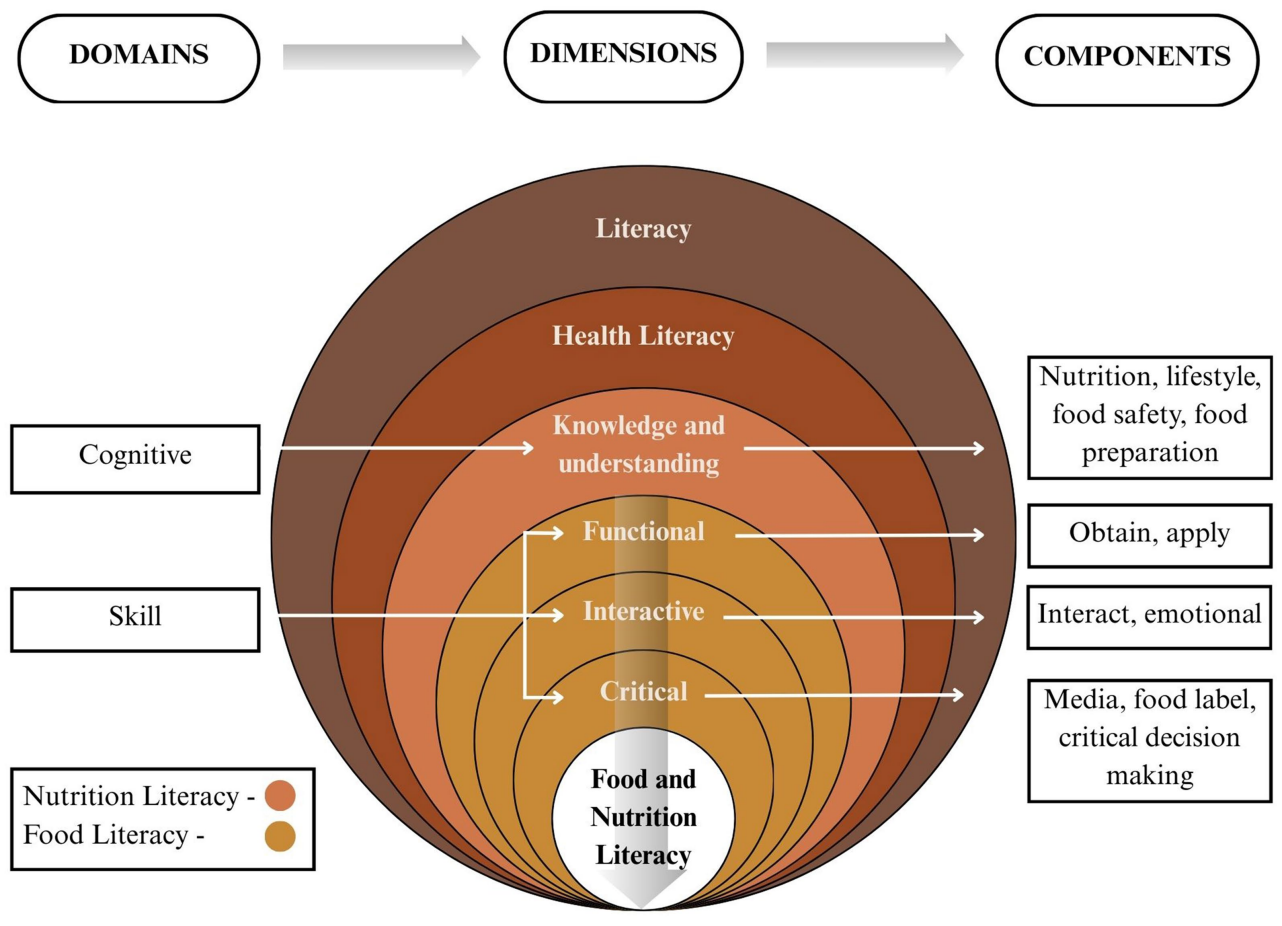
Conceptual framework of INFOLIT. Adapted from Doustmohammadian et al. (8), licensed under CC BY 4.0 and Zhang et al. (22), licensed under CC BY 4.0.

Building on these, the INFOLIT framework was conceptualized across two key domains - cognitive and skill—with four hierarchical dimensions: *Knowledge and Understanding*, *Functional*, *Interactive*, and *Critical* (Figure 1) (7, 8, 21, 22). Together, they encompass 11 distinct components reflecting both the ‘knowing’ (NL) and the ‘doing’ (FL) aspect of literacy.

The cognitive domain (knowledge and understanding) focusses on theoretical comprehension through four components: (i) *Nutrition*, knowledge of nutrients, food groups, dietary guidelines, and deficiencies; (ii) *Lifestyle*, awareness of physical activity for non-communicable diseases (NCDs) prevention; (iii) *Food Safety*, awareness of common food adulteration practices, and (iv) *Food Preparation*, hygiene practices in food storage, handling, and cooking (7, 8, 21, 22).

The skill domain covers the remaining three dimensions (*Functional*, *Interactive*, and *Critical*). The functional literacy dimension includes: (v) *Obtain*, the ability to access nutrition information from credible sources, and (vi) *Apply*, using knowledge in daily food choices. The interactive literacy dimension includes: (vii) *Interact*, discussing and exchanging knowledge about healthy eating habits with peers/family members; and (viii) *Emotional*, self-regulatory ability to resist cravings or temptations. Finally, the critical literacy comprises: (ix) *Media Literacy*, the capacity to deconstruct marketing claims, (x) *Food Label Literacy*, the ability to read and interpret packaged food labels, and (xi) *Critical Decision-Making*, the ability to critically analyse and make informed choices despite promotions/discounts (7, 8, 21, 22).

### Study design

Our study employed a cross sectional, sequential, exploratory mixed methods design, wherein a qualitative phase informed the subsequent quantitative phase (7, 21–24). Although the overall data collection was cross-sectional, this design was particularly appropriate for developing and validating measurement tools. It allowed us first to build a strong theoretical and conceptual foundation, and then empirically test the items for reliability and validity. The study was carried out in five different phases: (i) development of the tool (ii) assessing content validity (iii) assessing construct validity (iv) assessing reliability, and (v) determining cut off scores. The study was conducted in Hyderabad, Telangana State, India. The city, Hyderabad, was divided into 3 zones namely: old Hyderabad (zone 1), new Hyderabad (zone 2) and Secunderabad (zone 3). The study site was Hyderabad based randomly selected schools catering to middle-income category students from each zone. Middle-income schools were chosen as study sites because this group represents the dominant income class in developing nations such as India, thereby providing a context that is broadly reflective of the majority population. Government schools catering to lower income groups might have required greater simplification of items, while international schools may not have captured experiences relevant to the wider adolescent population. By focusing on middle-income schools, we aimed to develop a tool that balances contextual relevance with broader applicability. The inclusion criteria required participants to be Indian nationals in the age group 13 to 15 years, enrolled in school, and willing to provide consent. Exclusion criteria ruled out adolescents outside the specified age range, foreign nationals, those with chronic diseases, individuals following specialized diets under professional supervision, and those unable or unwilling to complete the survey.

**Figure 2:**
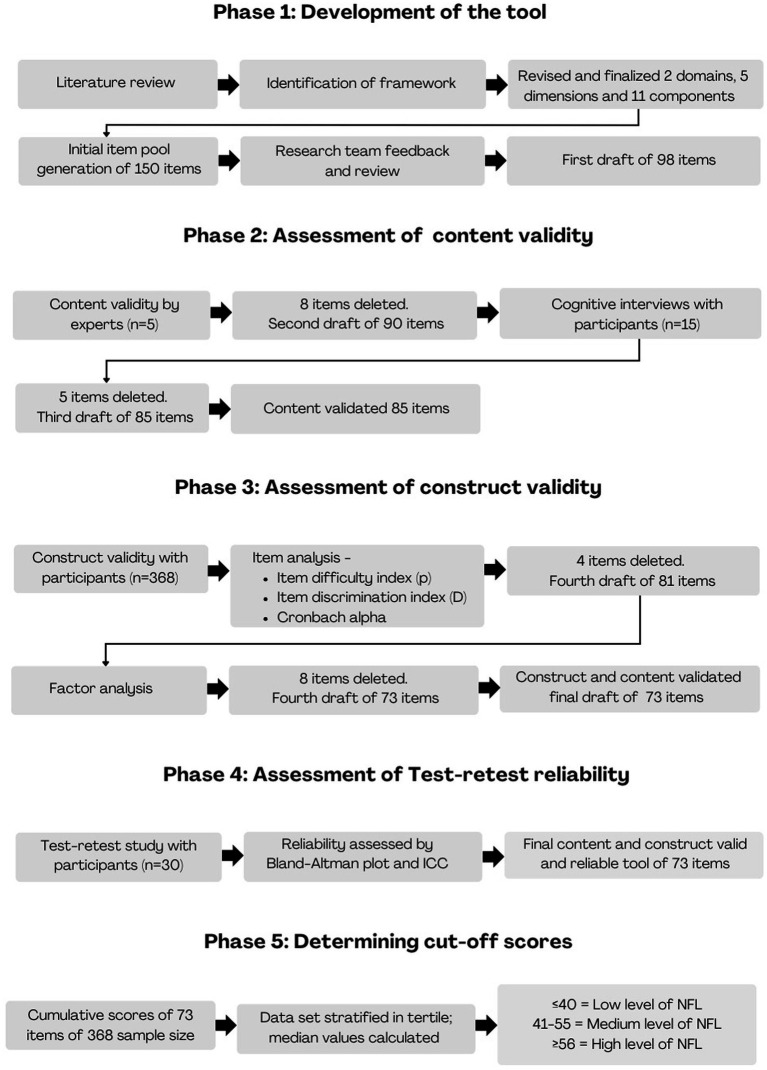
Development of the tool.

Adolescents aged 13–15 years fall into the middle adolescence phase, marked by growing cognitive abilities, emotional development, and increasing autonomy in decision-making, particularly in health-related behaviors like food choices (25, 26). In contrast, early adolescents (10–12 years) often lack the cognitive maturity to engage meaningfully with complex nutrition concepts, while late adolescents (16–18 years) exhibit more adult-like decision-making, shaped by established habits (27). Thus, the 13–15 age group represents a crucial developmental window to assess and improve NFL before patterns are fully established (28, 29). The detailed methodology for each phase is explained below (Figure 2).

#### Phase 1: Development of the tool

##### Identification of conceptual framework of NFL

The conceptual framework of NFL among adolescents followed a narrative approach. Peer-reviewed studies published in English were included, while theses, dissertations, unpublished data, and non-English papers were excluded. Studies focusing on nutrition literacy, food literacy, nutrition knowledge, or development of nutrition and food literacy tools were considered. Screening was continued until no additional relevant dimensions or constructs emerged, indicating saturation. An extensive literature search was carried out using terms like “food literacy,” “nutrition literacy,” “nutrition literacy tool,” and “nutrition knowledge” across English-language databases such as Google Scholar, PubMed, ScienceDirect, and Web of Science as it is difficult to translate the language specific research papers. Additionally, a backward search was done by examining reference lists of relevant articles and books, particularly those with titles containing “nutrition” or “food literacy” (6–9, 14–24, 30).

##### Item pool generation

Following the phase one framework and reviewing of existing literature 150 initial NFL questions were developed (5–8, 14–16, 21–24, 31–33). Of the 150 initial items (draft 1) developed, the pool was refined to 98 items (draft 2) prior to content validation. Items were deleted if they were repetitive, ambiguous, or overlapping, if they contained technical terms inappropriate for adolescents, if they were too lengthy or impractical, or if they did not align with the predefined framework dimensions. In addition, items irrelevant to the Indian cultural and geographical context were also excluded. The item pool consisted of two sections: (i) Socio-demographic details, including identification number, name, age, gender, class, religion, and educational background of both the participant and their parents, along with self-reported health status and (ii) the second section comprised the INFOLIT questions.

#### Phase 2: Content validity by experts

Content validity refers to how well an assessment tool encompasses an adequate range of items related to the construct being assessed (34). To ensure the validity of the items, five professionals with expertise in medicine (*n* = 1), sociology (*n* = 1), nutrition (*n* = 1), psychology (*n* = 1) and dietetics (*n* = 1), were consulted either face-to-face or through email correspondence. They assessed content validity using a structured form, where each question was rated on a 4-point Likert scale based on relevance, clarity, simplicity, and ambiguity (34). The CVI for each item was calculated by assigning a score of 1 if an expert rated the question as 3 or 4, and 0 if rated as 1 or 2. The total score given by the five experts was summed (e.g., 1 + 1 + 1 + 0 + 0 = 3) and divided by the number of experts (CVI = total score/total experts; e.g., 3/5 = 0.6). Items with a CVI of ≥0.8 were retained, while those below 0.8 were removed (34, 35). In the content validity scoring form, a separate row for suggestions of each question was provided for qualitative assessment of each question. Based on the comments received in content validity form, required changes were made in the tool.

##### Cognitive interviews with the participants

CIs were conducted to assess the clarity and effectiveness of questions by examining participants’ thought processes while answering (36). Fifteen participants meeting the study’s inclusion criteria were selected and they completed the tool’s question. The participants were selected using convenient sampling from one zone of Hyderabad, which was considered appropriate given the exploratory purpose of cognitive interviews, where the focus is on refining item clarity and comprehension rather than ensuring representativeness. Each interview lasted 30–45 min and followed a structured seven-step probing process (Table 1).

**Table 1:** Examples of the probing of the cognitive interview.

S.no.	Steps	Example
1.	Think-aloud/read aloud	“Can you read out question number 5 for me?” E.g. Participant X: Anemia is caused due to the deficiency of which mineral?
2.	Interpretation	“Can you tell me what this question is asking?” E.g. Participant X: This question is asking that anemia, i.e., decreased level of hemoglobin is caused due to which nutrient?
3.	Paraphrasing	“Can you repeat the question in your own words?” E.g. Participant X: Due to which nutrient deficiency, anemia happens?
4.	Recall	“How do you know what this question is trying to ask?” E.g. Participant X: During a school assembly, several girls fainted, prompting our physical education teacher to address the issue of anemia. He explained that low hemoglobin levels could lead to dizziness. We were instructed to include iron-rich foods in our diet to enhance hemoglobin levels and combat anemia.
5.	Confidence judgment	“How sure are you about what question is asking?” E.g. Participant X: I’m very much sure because of the school’s assembly.
6.	Response	“What option did you tick and why?” E.g. Participant X: I know Anemia is cause due to low Iron in the body.
7.	Cognitive debriefing	“Over all how was this tool from your perspective?” E.g. Participant X: It was easy to interpret and understand, except for a few words.

##### Scoring

The tool followed a structured format with closed-ended questions, including Likert-type, true/false, and dichotomous items. Responses were assessed using a binary scoring system, assigning 1 for behaviors aligned with NFL and 0 otherwise. Even Likert-type responses like “sometimes” were scored as 0 to emphasize the importance of consistent, daily actions. For example, a question on food safety asked whether restaurant cleanliness and staff hygiene mattered when dining out as sometimes was graded as 0. Similar scoring applied to items on personal practices and quality symbols. The decision to code “sometimes” as 0 was intentional, as the aim of INFOLIT is to assess consistent application of nutrition and food literacy practices. Occasional or partial behaviors do not indicate sufficient literacy, and thus were not awarded a positive score.

Four questions were excluded from scoring as they focused on attitudes, gathering data on food consumption frequency, media perceptions, and dietary information sources.

#### Phase 3: Assessing the construct validity of the tool

Construct validity refers to how well a test measures the theoretical construct it is designed to evaluate (37).

##### Sample size

Based on studies by Moitra et al. (38) and Rao et al. (39) considering 40% prevalence of nutritional knowledge, 95% confidence interval, 5% absolute precision, the sample size determined for pre-testing the tool was 369. Considering 10% dropout rate, a total of 400 participants were determined as an adequate sample size {*n* = Z^2^*p*(1-p)/ E^2^; n = the necessary sample size, Z = 1.96 (z-score linked to the targeted confidence level, which is 95%), *p* = 0.40 or 40% (anticipated proportion within the population), E = 0.05 or 5% (desired acceptable margin of error)}.


$$n=\frac{Z^{2}\times p\times 1-p}{E^{2}}$$

##### Administration of the tool

To assess construct validity, school visits were scheduled from August 2023 to September 2023 to administer the content validated tool among 400 adolescents aged 13–15 years. The INFOLIT, a pen-paper based self-administered tool, was distributed to participants seated in a classroom setting, with each classroom comprising of 30 participants. A clear guidance was provided on how to accurately complete the tool and fields required. They were reassured that their responses would not be incorporated into their individual report cards, emphasizing that the data collected was solely intended for research purposes. The tool was filled out by participants under the researcher’s supervision. The researcher addressed any participant doubts about readability or comprehension without suggesting or revealing the correct responses. The typical duration for finishing the tool ranged from 35 to 40 min.

### Item analysis

Item analysis is the process of evaluating test questions to determine their quality, effectiveness, and contribution to overall test performance. It involved three key aspects:

#### Item difficulty

Item difficulty helps compare the difficulty of different test items across various participants and examinee groups (40). The scores of participants (*n* = 368) were ranked in descending order and divided into three groups: the top 25% (*n* = 92), the bottom 25% (*n* = 92), and the middle 50% (*n* = 184). The highest and lowest scorers’ data were separated, and their scores were summed individually. The item difficulty index (DIF I or p) was calculated using the formula *p* = (H + L) / N, where H denotes the number of right answers within the group of highest scorers, L indicates the number of right answers in the group of lowest scorers, and N represents the total number of participants in both groups, including those who did not respond. An item is classified as difficult if *p* < 0.25, as easy if *p* > 0.80, and as not applicable if the value falls between 0.80 and 0.25 (41).

#### Item discrimination

Item discrimination assesses the proportion of high scorers versus low scorers who respond correctly to an item (42). The Discrimination Index (DI) was calculated using the formula DI = (H - L) / N, where H, L, and N represent the same values as previously defined. Items were classified based on the DI as follows: very good (DI > 0.40), reasonably good (DI = 0.30–0.39), marginal (DI = 0.20–0.29), and poor (DI < 0.19) (43).

#### Internal consistency

Internal consistency assesses how well the items within an instrument measure different facets of the same underlying construct (44) Cronbach’s alpha is commonly used to quantify internal consistency by evaluating how closely related a set of items are. The value generally falls between 0 and 1, where higher values suggest greater internal consistency (45).

### Factor analysis

Factor analysis is a statistical method employed to identify the underlying relationships between variables by grouping them into latent constructs or factors. It is mainly used to reduce data dimensionality and identify patterns within large datasets without losing the important information (46–49). A critical aspect of factor analysis is the concept of eigenvalues, which represent the amount of variance accounted for by each factor extracted from the dataset. Specifically, an eigenvalue quantifies the proportion of total variance that a particular factor explains. To visualize and determine the appropriate number of factors to retain, researchers often employ the scree plot, which graphs the eigenvalues in descending order against the number of factors. The scree plot is instrumental in identifying a “break” or “elbow” point, suggesting where the addition of more factors begins to contribute minimal additional variance (50).

To further refine the item pool and understand underlying relationship between variables, factor analysis was employed for the cognitive and skill domains. The numbers of factors were determined through an examination of eigenvalues, a scree plot, and the percentage of cumulative variance explained by each factor. Questions that did not exhibit any factor loading were identified for removal from the item pool, contributing to a more refined set of items for analysis (51).

#### Phase 4: Assessing test–retest reliability of the tool

Reliability is widely regarded as the key indicator of a test’s accuracy. It reflects how consistently and dependably a test measures, with minimal influence from random errors or fluctuations over time. The 73-item pool was administered twice among 30 participants (*n* = 15 girls; *n* = 15 boys) to test the stability of the tool over time, who had not participated in the study so far to ensure that the test content was not revealed. The participants were not informed about the retest, which occurred 2 weeks after the initial measurement. The variations between the two measurements were graphed against the averages of the two measurements using the Bland–Altman approach (52). Furthermore, we used, ICC which quantifies the degree of agreement or reliability between measurements made by different observers or instruments, particularly concerning continuous data or ratings. It reflects the proportion of total variance in a set of measurements that can be attributed to the variability between subjects rather than the variability within subjects (53, 54).

#### Phase 5: Determining cut-offs for INFOLIT using a tertile-based stratification method

INFOLIT cut-off scores were determined through a systematic process that validated 73 items after both item and factor analyses, leading to the deletion of 4 and 8 items, respectively. The cumulative score of these 73 items was calculated for each of the 368 observations in the sample. After computing cumulative scores, the dataset was divided into tertiles. This stratification showed a score distribution ranging from a minimum of 19 to a maximum of 66 out of 73 items. To improve categorization accuracy, median values were calculated for each tertile. Median values were used because, unlike the mean, which was influenced by extreme values, the median was less sensitive to outliers and provided a more stable measure of central tendency. This approach helped define the distinct levels of NFL within the sample more precisely.

### Ethical statement

The research plan received approval from the Institutional Ethics Committee (IEC) at ICMR- National Institute of Nutrition, Jamia-Osmania, Hyderabad, with the protocol number 18/III/2023. Permission for conducting study was taken from the school’s principal. Signed consents forms from participants and their parents were obtained for all the three phases (CIs, pre-testing, and test–retest reliability). Study and institute background details were shared well in advance to the school, parents and participants. The study prioritized privacy and maintained a strict confidentiality of personal information for both schools and participants.

### Statistical analysis

Various item-level analyses p, D and CVI were performed using Excel. Internal consistency was performed using Cronbach’s *α*, and the underlying structure of observed variables was done through factor analysis, both conducted using SPSS 27.0. Factor loading and item level analyses and CVI were used to keep or drop items. To examine the test–retest reliability, the ICC was calculated using SPSS 27.0. Furthermore, the construction of a Bland–Altman plot using R Software 4.2.1 provided a visual representation to assess agreement between test–retest score.

## Results

### Phase 1: Development of a tool

#### Identification of conceptual framework of NFL

A total of 27 studies were obtained from the literature search, of which 13 studies were included in the review (6–8, 14–16, 21–24, 31–33). Of these, three studies by Doustmohammadian et al. (8), Khorramrouz et al. (7), and Zhang et al. (22) addressed both NL and FL definitions and their components. Based on these three studies and the Nutbeam’s model of health literacy a conceptual framework was finalized. It included two domains with four dimensions and eleven components (20). Two domains identified were: (i) Cognitive domain and (ii) Skill domain. First domain, i.e., cognitive domain included one dimension, namely: (a) Nutrition and food knowledge and its understanding with four components (nutrition knowledge, lifestyle, food safety, food preparation). Second domain, i.e., skill domain is based on health model of Nutbeam and levels of HL which included three dimensions (b) Functional literacy with two components (apply and obtain) (c) Interactive literacy with two components (interact and emotional), (d) Critical analysis of the information with three components (media, critical decision making and food label).

#### Item pool generation

A pool of 150 items was generated at the first phase of the study. Following the process of eliminating and consolidating redundant items, the refined item pool was ultimately streamlined to consist of 98 items with 51 in cognitive and 47 in skill domain items. The type of items included were (i) 3-point Likert-type (ii) multiple choice and (iii) true false. Few questions like food frequency, sources of diet related information, ability to judge media related diet information were also added to understand their attitude and practice about nutrition and food (33). These questions were excluded from the scoring.

### Phase 2: Content validity by experts

Based on CVI scores received <0.8, seven questions were deleted. As per advice of the panel, questions containing technical terms or jargons were either simplified or removed. Redundant questions falling under various components were deleted. The tool obtained after CVI score and expert comments had 90 items with 48 in cognitive and 42 in skill domain.

#### Cognitive interviews with the participants

Following the CIs, five more questions were removed from skill domain and 11 items were modified due to their perceived complexity, redundancy, lack of relevance among the target age group. The final content validated item pool tool consisted of 85 items with 43 in cognitive and 42 in skill domain.

### Phase 3: Assessing the construct validity of the tool

Although 400 participants were selected for the administration of the but 32 were excluded from the study due to their unavailability on the designated study day and few did not meet the inclusion criteria. The average age of participants was 13.79 ± 0.54 years out of which 51.08% were male and 48.9% were female.

The p ranged from 0·10 to 0·98 with 25 easy, 6 difficult questions and all the rest (54) being in the desirable range of difficulty (Table 2). The D ranged from 0.00 to 0.88, with 39 items being classified as very good items with D values greater than 0.40, 15 items being categorized as reasonably good items with D values ranging from 0.30 to 0.39, 18 items were considered marginal with D values between 0.20 and 0.29, and 13 items were labeled as poor items with D values less than 0.19 (Table 2). Cronbach alpha value obtained for overall 85 items was 0.81 which was considered good (Table 2). Based on item analysis, four items were deleted and 23 items were modified and rephrased. The tool now consisted of 81 items with 40 items in cognitive and 41 in skill domain (Table 3).

**Table 2:** Item difficulty, item discrimination and internal consistency results.

Result of item analysis	Number of items (*n* = 85)
Item difficulty index (*p*); overall range = 0.10 to 0.98	
Easy items (>0.80)	25
Desirable items (0.24–0.79)	54
Difficult items (<0.25)	6
Item discrimination (D); overall range = 0.00 to 0.88	
Very good items (>0.40)	39
Reasonably good items (0.30–0.39)	15
Marginal items (0.20–0.29)	18
Poor items (<0.19)	13
Cronbach alpha (α); acceptable value >0.70	Value
Overall Cronbach alpha of 85 items (α)	0.81

**Table 3:** Total questions deleted during content and construct validity.

Domains	CVI	Cognitive interviews	Item analysis	Factor analysis	Total questions deleted	Final question number
Cognitive	3	5	3	3	14	51–14 = 37
Skill	5	-	1	5	11	47–11 = 36
Total	8	5	4	8	25	98–25 = 73
	98–8 = 90	90–5 = 85	85–4 = 81	81–8 = 73		73 items

#### Factor analysis

**Figure 3:**
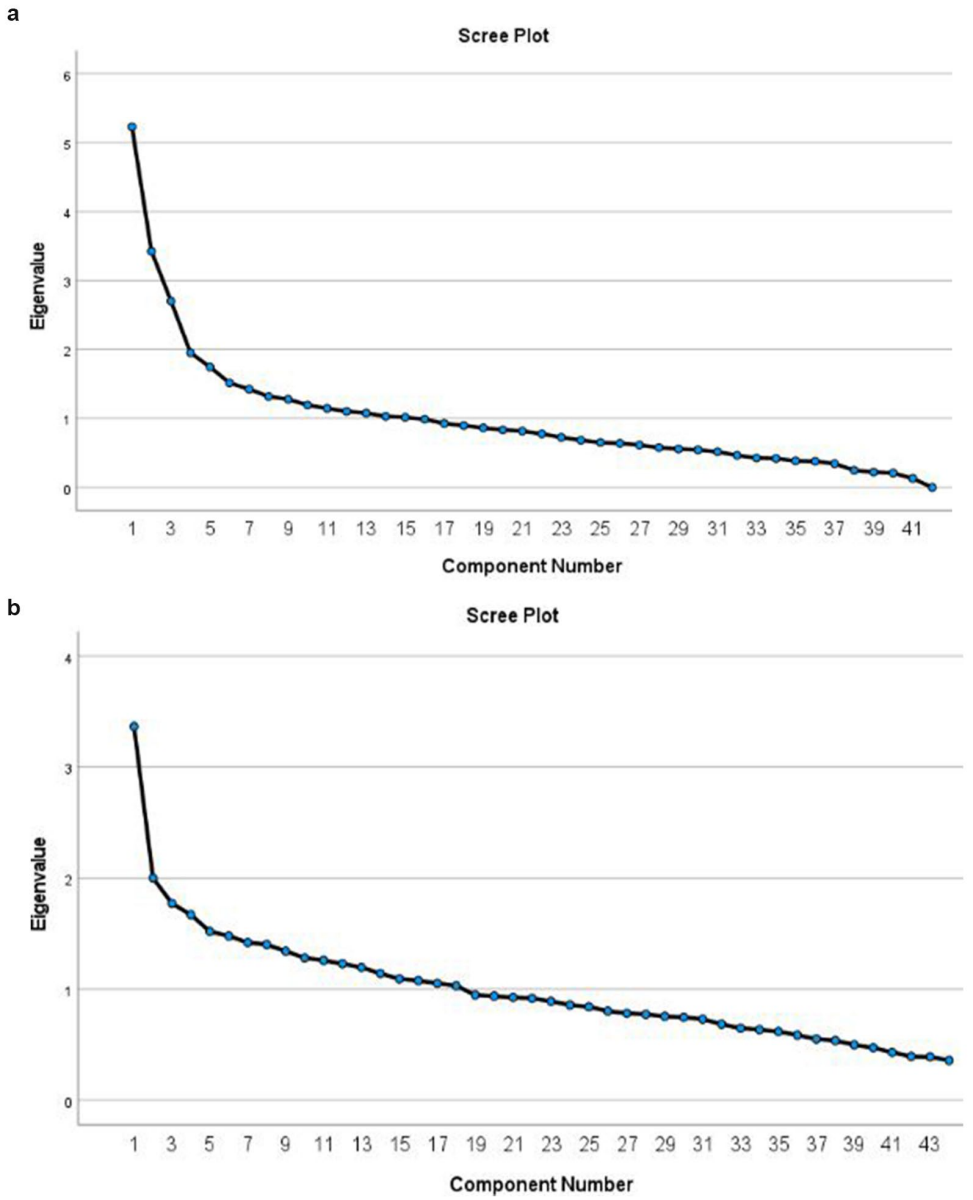
Scree plot for the domains. **(a)** Cognitive domain and **(b)** Skill domain.

In our study, the scree plots for the cognitive (Figure 3a) and skill (Figure 3b) domains exhibited a clear bend at five factors, indicating that five components should be retained for further analysis. The eigenvalues obtained for the fifth component were 1.52 (cognitive) and 1.75 (skill). Eigenvalues reflect the amount of information or variance a factor explains values above 1 are generally considered significant (55, 56). This suggested that each of the five factors in the current study (represented as F1, F2, F3, F4, F5) captured a meaningful amount of the data, supporting a five-factor solution.

The cumulative variance explained by the five components was 23.47% for the cognitive domain and 35.82% for the skill domain. This means that the five components together explained around one-fourth to one-third of all the differences in participants’ responses. In literacy and behavior-related research, where human factors are complex and multi-layered, this level of explained variance is considered acceptable (55–57). The emergence of five distinct components supports the conceptual idea that NFL is multi-dimensional and need to be assessed across different layers.

During the analysis, it was found that three questions from the cognitive domain and five from the skill domain had no factor loading values, so they were excluded. In the cognitive domain, Factor F1 showed its highest loadings (≥0.4) for items 18.1, 18.2, and 18.8, which assessed knowledge of healthy and unhealthy foods (refer Supplementary material for respective questions). Factor F2 also had strong loadings (≥0.4) for items 18.9, 18.4, 36.1B, and 18.7, focusing on understanding junk food and interpreting symbols on food labels. In the skill domain, Factor F1 demonstrated very high loadings (≥0.7) for items 34.7, 34.6, 34.8, 34.9, and 34.5, which related to the practice of checking food labels for sugar, salt, fat, and ingredients. For Factor F2, all items were strongly associated with loading values of ≥0.7.

Due to the limited sample size and the exploratory nature of the analysis, factor analysis was primarily used for item reduction rather than confirming a definitive factor structure. Construct validity was supported through content validation by experts and assessment of internal consistency, ensured that the items adequately capture the multidimensional nature of NFL.

### Phase 4: Assessment of test–retest reliability

**Figure 4:**
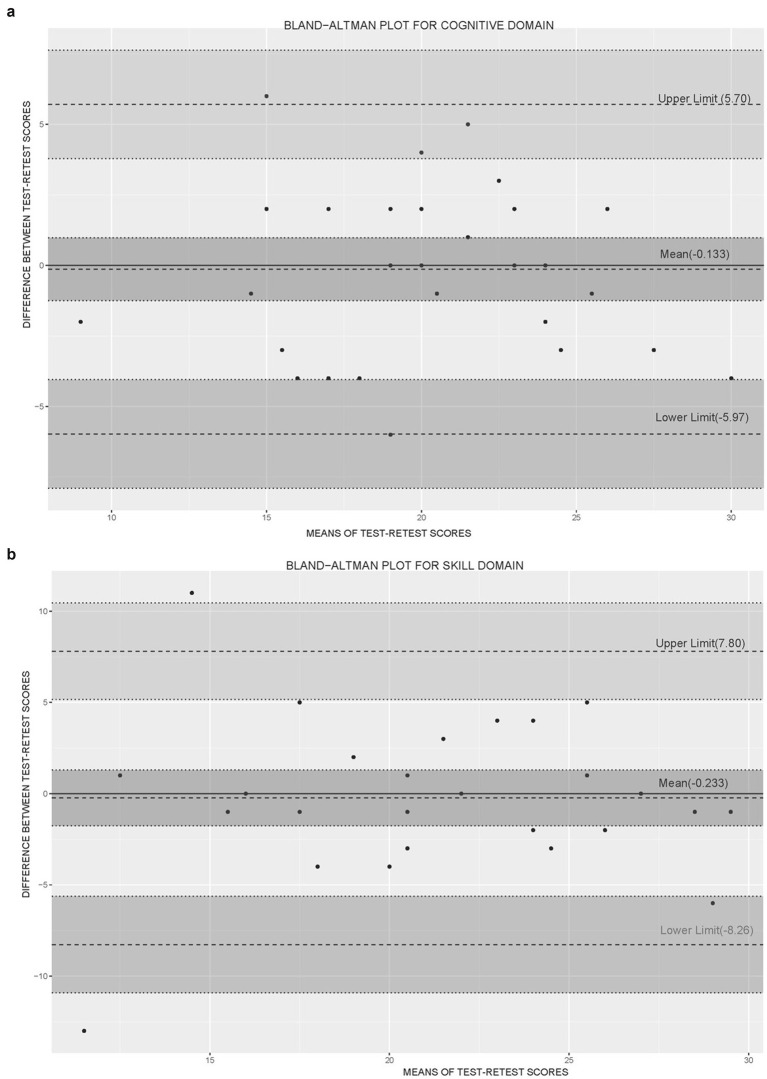
A Bland–Altman plot for domains. **(a)** Cognitive domain and **(b)** Skill domain.

The Bland–Altman plot showed minimal mean bias (−0.133 for cognitive and −0.233 for skill domains) (Figure 4), indicating good agreement between test and retest scores, despite a few outliers. The ICC for overall scores was 0.895, confirming very good reliability (52). The ICC reflects how consistent responses are over time, with values above 0.75 considered good (53). This suggests the INFOLIT tool yields stable and repeatable results.

### Phase 5: INFOLIT cut-off scores

On a scale of 73 items, anyone scoring ≤40 is considered to have low level of NFL, whereas anyone scoring between 41 and 55 is considered to have medium level of NFL. Similarly, anyone scoring ≥56 is considered to have high level of NFL (see Table 4).

**Table 4:** INFOLIT categorization of scores.

Score range (out of 73)	NFL Level
≤ 40	Low
41–55	Medium
≥ 56	High

## Discussion

The current study utilized a five-phase cross-sectional, sequential exploratory mixed methods design. INFOLIT serves as a useful evaluation instrument and can play a role in shaping public health nutrition policies.

It is important to note that NFL encompasses more than basic knowledge and understanding of food and nutrition, it also includes the ability to apply NFL concepts (functional literacy), interact with friends, family, and the immediate environment (interactive literacy), and critically evaluate food label claims to make healthy choices (critical literacy) (14). This study presents a comprehensive framework of NFL, aligned with Nutbeam’s model of health literacy and the Doustmohammadian framework of food and nutrition in children (8, 20). Most studies focus only on specific dimensions of NFL, such as food label literacy (18), menu board literacy (17) or concentrate solely on constructs related to NL and FL (58). Only a few studies have attempted to capture the full scope of NFL by integrating both nutrition and food literacies (7, 14). Conceptually, the tool also contributes by reframing adolescent NFL as a developmental capacity rather than merely a behavioral outcome. This perspective highlights the importance of equipping young people with critical and interactive literacies that enable them to navigate marketing claims, peer influences, and rapidly changing dietary landscapes dominated by ultra-processed foods. By positioning adolescents as active agents within their food environments, INFOLIT strengthens the theoretical foundation for designing interventions and policies that go beyond knowledge dissemination toward cultivating practical, interactive, and critical skills necessary for lifelong healthy dietary behavior.

A systematic review emphasizes that NFL is highly contextual, shaped by food environments, dietary behaviors, culture, and socioeconomic status (59). Existing tools developed across various countries are often population-specific, underscoring the necessity for culturally valid and contextually appropriate instruments. In this context, INFOLIT has been developed specifically for Indian adolescents, incorporating items aligned with Indian dietary guidelines, prevalent food habits, and marketing claims relevant to the Indian food environment (2, 60).

While prior studies reported scale-level CVI (CVI-S) values of 0.94 to 0.92, our study prioritized item-level CVI (I-CVI) for a detailed assessment of individual items (23, 24, 43, 44). Most items had an I-CVI value >0.8, which is consistent with findings from other studies (6, 24). This approach was particularly appropriate given the tool’s multidimensional structure, ensuring each item effectively represented its domain. According to Lynn’s criteria, a minimum of three experts is essential for content validity, with five to ten experts recommended to balance diversity and manageability (61). In alignment with these guidelines, five experts from diverse domains like psychology, medicine, sociology, clinical nutrition, and dietetics were consulted to ensure that the content was both interdisciplinary and culturally appropriate. This aligns with studies such as the Preschool-FLAT, which also used five experts and reported a CVI of 0.94, demonstrating that small but varied panels can yield high agreement (16). In contrast, tools such as M-FNLIT by Khorramrouz et al. (24) FNLAT by Ashoori et al. (6), and TFLAC by Amin et al. (58) used Delphi approach with larger panels (16–20 experts) and multiple rounds, often resulting in similar higher CVI values (0.92–0.98). However, while Delphi methods can enhance consensus, they are resource and time-intensive. The single-round CVI process in our study provided both quantitative cut-offs and qualitative feedback in a more time-efficient manner. Furthermore, our emphasis on I-CVI rather than CVI-S helped identify individual items requiring modification, rather than masking them under an overall score, and the inclusion of culturally relevant examples likely contributed to the higher relevance ratings observed for certain items.

Very few studies to date have pre-tested their tools with the target population before assessing construct validity. Previous researchers have often used qualitative methods for pre-testing like CIs and focus group discussions, where participants are shown individual items and asked to share their thoughts on each (6, 15, 17, 21). These qualitative approaches are valuable because they not only provide feedback on item clarity but also reveal unexpected issues with specific questions (62). This approach allows for early identification of items that are confusing, culturally irrelevant, or too technical for target group. Similarly in our study, CIs revealed that participants generally understood the importance of foods and could relate them to their specific functions. However, this was not always the case when asked directly about the role of macronutrients except for protein, which was more familiar due to its popularity. For instance, students did not initially associate carbohydrates with energy. When examples such as wheat, rice, and millet were added as sources of energy, they were better able to make this connection. The use of CIs not only supported simplification of language but also ensured that the tool captured NFL in a way that was meaningful and understandable to the intended age group. This step helped strengthen the overall content validity of the tool in a real-world context.

In psychometric validation studies, various approaches are available for item refinement, including Classical Test Theory (CTT) based statistics such as item difficulty, discrimination, and internal consistency, as well as Item Response Theory (IRT) models such as the Rasch model (6, 15, 16, 18, 24). While Rasch and other IRT methods offer detailed item-level diagnostics, they typically require large, diverse samples and advanced statistical modelling, which may not be feasible during the early stages of tool development or in resource-limited contexts. For these reasons, our study adopted a CTT-based approach, which provides clear and interpretable metrics for identifying items that are too easy, too difficult, or poor at differentiating between high and low performers. In our analysis, the item difficulty index (p) ranged from 0.10 to 0.98, and the item discrimination ranged from 0.00 to 0.88. Consistent with similar studies, items with a difficulty index below 0.30 were considered for removal, although those deemed essential by expert reviewers were revised rather than discarded (63). Unlike other studies that have reported mean difficulty and discrimination at the overall scale level, we focused on item-level analysis to enable targeted improvements to individual items (64).

Consistent with previous research, items with low or non-significant factor loadings were removed (63). Item refinement in our study combined both item analysis and factor analysis. Although factor analysis did not reveal clear underlying patterns due to sample size limitations, it still supported item reduction and strengthened the tool’s structure. Limited number of research has utilized both the analysis for item reduction.

Previous studies have reported overall internal consistency using Cronbach’s alpha, with values ranging from 0.77 to 0.90 (16, 24, 63, 65). INFOLIT also showed high internal consistency (*α* = 0.81), which aligns with these findings. Only a few studies have reported test–retest reliability, typically using the ICC, with values ranging from 0.64 to 0.93, indicating moderate to excellent reliability (16, 18, 23). Our study assessed test–retest reliability using both ICC and the Bland–Altman plot. INFOLIT reported an ICC score of 0.95, reflecting excellent reliability and strong agreement between test and retest responses. In addition, the Bland–Altman plot showed minimal mean bias, further supporting the reliability of the tool.

To enhance user-friendliness, the tool was tailored for self-administration. The focus on adolescents in grades 8 to 10 was strategic, recognizing this age group’s pivotal stage in developing food-related behaviors and skills. An important decision in the scoring system was to assign a score of 0 to responses such as “sometimes.” This scoring approach strengthens the tool’s ability to distinguish between partial knowledge and comprehensive literacy. The study’s geographic scope was limited to the Hyderabad region, which may restrict the generalizability of the findings to other parts of India. The relatively small sample size constrained our analysis to item reduction using exploratory factor analysis, and confirmatory factor analysis (CFA) could not be conducted to validate the underlying factorial structure. In addition, the study’s focus on middle-income adolescents combined with the use of convenience sampling may limit the findings of the research, as participants may not fully represent the diversity of the broader adolescent population. Furthermore, response and recall bias, inherent in self-report tools, is another limitation. Future research should include adolescents from diverse socioeconomic backgrounds and regions, utilizing larger and more representative samples, to enhance the empirical validation of the framework. Nevertheless, the consistent test–retest reliability and internal consistency observed in this study support the dependability of the tool.

### Future implications of the research

The availability of a validated tool like INFOLIT offers critical opportunities for health educators and policymakers. It can be used to: (i) inform the design of structured and age-appropriate nutrition education curricula, (ii) guide the development of measurable, evidence-based nutrition intervention programs, and (iii) enable objective assessment of program impacts. By identifying specific gaps in adolescents’ nutrition and food literacy, INFOLIT facilitates targeted decision-making. Future studies with larger and more diverse samples should also undertake CFA to strengthen the construct validity of the INFOLIT framework.

## Conclusion

In conclusion, the development of the INFOLIT represents a significant step in addressing the critical issue of the absence of any validated and reliable tool among Indian adolescents. Beyond measuring levels of literacy, the tool can help identify specific gaps and guide the design of tailored nutrition education strategies. Its availability offers a practical framework for policymakers and educators to develop structured nutrition curricula, create evidence-based intervention programs, and objectively assess their outcomes. Future research should focus on conducting longitudinal studies to examine the tool’s stability over time, using it in intervention settings to evaluate its effectiveness in capturing changes in nutritional literacy, and performing cross-validation in other LMICs to test its generalizability and cultural relevance.

## Data Availability

The original contributions presented in the study are included in the article/Supplementary material, further inquiries can be directed to the corresponding author.
