# Supplementary material for: Development and Validation of an Indian Nutrition and Food Literacy Tool (INFOLIT) for adolescents
Source: Front Nutr. 2025 Nov 13;12:1626673. doi: 10.3389/fnut.2025.1626673 (PMC12671486; doi:10.3389/fnut.2025.1626673)
Supplement: Supplementary file 1 [file Supplementary_file_1.docx]

**ICMR - NATIONAL INSTITUTE OF NUTRITION (NIN)**

**INDIAN NUTRITION AND FOOD LITERACY TOOL (INFOLIT)**

Date: Time: School name:

**PART I: SOCIO-DEMOGAPHIC DETAILS:**

A. Name of the student: ___________________

B. Age: ___________________

C. Class: ___________________

D. Phone number: ­­­­­­­­­ ___________________

E. Gender: Male □ Female □ Prefer not to say □ Others, please specify________

F. Religion: Hindu □ Christian □ Muslim □ Sikh □ Jain □ Others □

E. Father’s education (tick the correct response):

- Illiterate□ Primary(1^th^-5^th^)□ Secondary (6^th^-10^th^)□ Senior secondary (11^th^-12^th^) □
- Undergraduate (B.Sc., B.Com, B.Tech, B.Pharma, BA, BBA etc.) □
- Postgraduate (M.Sc., M.Com, M.Tech, M.Pharma MA, MBA etc.) □
- Ph.D. and above □
- Others, please specify ___________________

G. Father’s occupation: ___________________

H. Mother’s education (tick the correct response):

- Illiterate□ Primary(1^th^-5^th^)□ Secondary (6^th^-10^th^)□ Senior secondary (11^th^-12^th^) □
- Undergraduate (B.Sc., B.Com, B.Tech, B.Pharma, BA, BBA etc.) □
- Postgraduate (M.Sc., M.Com, M.Tech, M.Pharma MA, MBA etc.) □
- Ph.D. and above □
- Others, please specify ___________________

I. Mother’s occupation: ___________________

J. Do you have any of the following conditions?

Diabetes □ Thyroid □ PCOS (Polycystic Ovarian Syndrome) □ Neurological disease □ None of the above □ I don’t know □ Others, please specify ___________________

K. Do you follow any specific diet under qualified nutritionist or dietitian?

Yes □ No □

If yes, please specify what type of diet it is, ____________________________

**PART II: MULTIPLE CHOICE QUESTIONS (MCQ)**

*There are total 38 questions in this booklet, please attempt all questions. Do not leave any question blank. In case of any query, please ask the guide. Do not discuss with others. Tick one option for each question. Wherever in front of question, it is mentioned, “more than one option can be correct”, there you can tick one or more than one option.*

**COGNITIVE DOMAIN**

1. A variety of foods such as cereals (wheat, rice, millets like ragi) and pulses (green moong dal, red gram dal), fruits and vegetables, milk or meat products or egg, nuts and oil should be consumed:

a. Daily

b. Alternate days

c. Once a week

d. Don’t know

1. As per Indian dietary guidelines, one should consume ________________ to maintain a healthy body.

i. variety of fruits and vegetables

ii. variety of cereals, pulses and millets

iii. variety of fats and oils

iv. variety of meat, milk and their products

Select the correct option:

a. i and ii

b. i, ii and iii

c. i, ii, iii and iv

d. Don’t know

1. Read the following statements and tick the appropriate answer:

3.1) Eating eggs boost immune system. a. True  b. False c. Don’t know

3.2) Cereals and millets are a major source of energy. a. True  b. False c. Don’t know

3.3) Fats and oils are important for healthy skin. a. True  b. False c. Don’t know

1. The richest sources of protein are:

a. Green leafy vegetables

b. Vegetable and fruits

c. Milk, pulses, meat and eggs

d. Don’t know

1. Eating food rich in fibre is good for heart health. The richest sources of fibre are:

a. Chicken and Egg

b. Vegetable and Fruit

c. Milk and Sugar

d. Don’t know

1. From the following options, which one do you consider as the best source of healthy fats?

a. Red meat (mutton, pork) fat

b. Mayonnaise

c. Almonds and walnuts

d. Don’t know

1. From the following options, which one do you think contains unhealthy fats? (more than one option can be correct)

a. Pizza

b. Fried chips

c. Veg noodles

d. Don’t know

1. To keep your heart healthy, good cholesterol is important. To increase good cholesterol in the body, one should:
   1. Eat chips/pizza/burgers
   2. Not exercise
   3. Eat fruits/nuts/fish
   4. Don’t know
2. How much of your plate should be filled with fruits and vegetables in a day?
3. Half of your plate
4. Quarter of your plate
5. Less than a quarter of your plate
6. Don’t know
7. Are you aware of ‘my plate for the day’ developed by ICMR- National Institute of Nutrition (NIN)?
8. Yes
9. No

If yes, how did you come to know about it? Please elaborate. ___________________________________________________________________________

1. Milk is important for building strong bones. Adolescents should consume at least

of milk per day.

1. 1 litre (1000 mL)
2. ½ litre (500 mL)
3. 250 ml
4. Don’t know
5. Anaemia, that is, decreased level of haemoglobin is caused due to deficiency of which mineral?
6. Iron
7. Calcium
8. Zinc
9. Don’t know
10. A rich source of Vitamin C among the following is
11. Guava
12. Cucumber
13. Buttermilk
14. Don’t know
15. As per the World Health Organization (WHO), an adolescent should drink at least

of water per day.

1. 1.5 – 2 litre
2. 1 litre
3. 5 litre (L)
4. Don’t know
5. Drinking or eating with meals increases the nutrients we get from the food we eat.

a. Tea or coffee

b. Carbonated beverages

c. Fruits like guava, papaya

d. Don’t know

1. Read the following food items and tick the correct response.

| **S.no.** | **List of food items** | **Healthy (a)** | **Unhealthy (b)** | **Don’t know (c)** |
| --- | --- | --- | --- | --- |
| 1. | Tomato salad |  |  |  |
| 2. | Sweetened yogurt |  |  |  |
| 3. | White bread |  |  |  |
| 4. | Packaged sweetened pista kesar milk |  |  |  |
| 5. | Paneer dosa |  |  |  |
| 6. | Tomato ketchup |  |  |  |
| 7. | Jackfruit ice-cream |  |  |  |
| 8. | Packaged sweetened fruit juice |  |  |  |

1. Read the following statement and tick the correct response.

| **S.no.** | **Statement** | **Agree (a)** | **Disagree (b)** | **Don’t know (c)** |
| --- | --- | --- | --- | --- |
| 1. | Calcium is the mineral responsible for strong bones and teeth. |  |  |  |
| 2. | Steaming and boiling are healthier ways of cooking than frying and grilling (tandoor). |  |  |  |
| 3. | Separate cutting boards should be used for raw meats and fresh vegetables. |  |  |  |
| 4. | Long nails do not lead to contamination of food. |  |  |  |
| 5. | Balancing diet and physical activity helps with weight management. |  |  |  |

1. Food adulteration is adding impure/cheap/harmful substances to food. Which of the following is an example of food adulteration? (more than one option can be correct)
2. Adding water in milk
3. Adding cow dung powder in red chili powder
4. Adding sugar in honey
5. Don’t know
6. The best way to wash fresh fruits and vegetables is:
7. with Regular soap
8. with Anti-bacterial sponge
9. under Running tap water
10. Don’t know
11. According to the World Health Organization (WHO), hands should be washed thoroughly for seconds before and after cooking and eating of food.

a. 10

b. 20

c. 60

d. Don’t know

1. To maintain food hygiene, cooked and uncooked foods in the fridge, should be stored

a. in same shelves of the fridge

b. in different shelves of the fridge

c. in the door side of the fridge

d. Don’t know

1. According to the World Health Organization (WHO), adolescents (10-19 years) should do at least minutes of physical activity every day.

a. 30

b. 45

c. 60

d. 120

1.
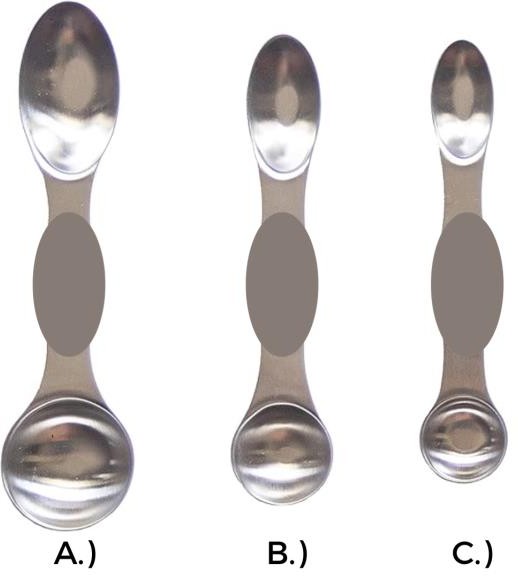
Adolescents’ daily salt consumption should not exceed (use the picture given below for reference).

a. A – 1 tablespoon (15 g/ day

b. B – 2 teaspoon (10 g/ day)

c. C – 1 teaspoon (5 g/ day)

d. Don’t know

1. Read the following question and tick the correct response about quality symbols.

| **S.no.** | **Picture of the symbol** | **24.1 – Have you seen this before** |
| --- | --- | --- |
| A. | 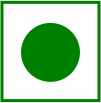 | a. Yes  b. No  c. Don’t know |
| B. | 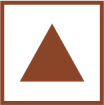 | a. Yes  b. No  c. Don’t know |

**SKILL DOMAIN**

24. Read the following question and tick the correct response about quality symbols.(contd.)

| **S.no.** | **Picture of the symbol** | **24.2 – Symbol name** | **24.3 – Is it a compulsory labeling for the packaged food?** |
| --- | --- | --- | --- |
| A. | 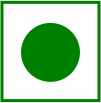 | a. Veg  b. Non-veg  c. Don’t know | a. Yes  b. No  c. Don’t know |
| B. | 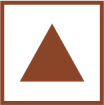 | a. Veg  b. Non-veg  c. Don’t know | a. Yes  b. No  c. Don’t know |

1. Read the following statement and tick the correct response.

| **S.no.** | **Statement** | **Agree (a)** | **Disagree (b)** | **Don’t know (c)** |
| --- | --- | --- | --- | --- |
| 1. | If food like burger and french fries are available at a cheaper price, it’s good to buy more. |  |  |  |
| 2. | Skipping meals (breakfast, lunch or dinner) is a good way to lose weight. |  |  |  |
| 3. | Regularly eating high fat foods (vada, bajji) and processed foods (pizza, chocolates, cookies) can raise the risk of cancer and heart attacks. |  |  |  |

1. On an average, how many times in a week, do you skip your breakfast?
2. Never (0 times in a week)
3. Rarely (1-2 times in a week)
4. Most of the times (3-5 times in a week)
5. Always (7 times a week)
6. How do you identify spoiled food? (more than one option can be correct)
7. Foul odour
8. Unusual appearance (mould growth, texture change)
9. Tastes bad, sour, or different from its usual Food Literacyavour
10. Don’t know
11. Tick the following options based on your personal practice.

| **S.no.** | **Statement** | **Never (a)** | **Sometimes (b)** | **Always (c)** |
| --- | --- | --- | --- | --- |
| 1. | When dining in a restaurant, is the cleanliness of the restaurant and the staff important to you? |  |  |  |
| 2. | Are you willing to learn more about nutrition information? |  |  |  |
| 3. | Do you take the initiative to spread nutrition knowledge with others? |  |  |  |
| 4. | Do you talk about healthy food at home? |  |  |  |
| 5. | Do you find yourself craving and consuming junk foods such as ice cream, cookies, chips etc. at midnight? |  |  |  |
| 6. | When you’re sad or feeling low, do you quit or stop eating? |  |  |  |
| 7. | Following any fight, argument, disagreement, or during times of low mood, do you tend to consume chocolates, chips, or other snacks? |  |  |  |
| 8. | If your favorite celebrity or star comes in a chocolate advertisement, would you buy that chocolate? |  |  |  |
| 9. | Do you check a label on the food packet you purchase? |  |  |  |

1. What do you check in packets while buying food? Tick your response.

| **S.no.** | **Statement** | **Never (a)** | **Sometimes (b)** | **Always (c)** |
| --- | --- | --- | --- | --- |
| 1. | Manufacturing date |  |  |  |
| 2. | Expiry date |  |  |  |
| 3. | Best before |  |  |  |
| 4. | Manufacturing address |  |  |  |
| 5. | Ingredients list |  |  |  |
| 6. | Amount of fat |  |  |  |
| 7. | Amount of sugar |  |  |  |
| 8. | Amount of salt/ sodium |  |  |  |
| 9. | Amount of cholesterol |  |  |  |
| 10. | Veg/ Non-veg symbols |  |  |  |
| 11. | Health claims (e.g. oats are good for heart health) |  |  |  |
| 12. | Allergen (e.g. contains peanut which can lead to allergic reaction to some) |  |  |  |

1. Given below is the picture of fruit juice and its nutrient label. Read the nutrient label carefully and answer the following questions.


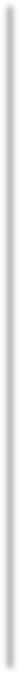


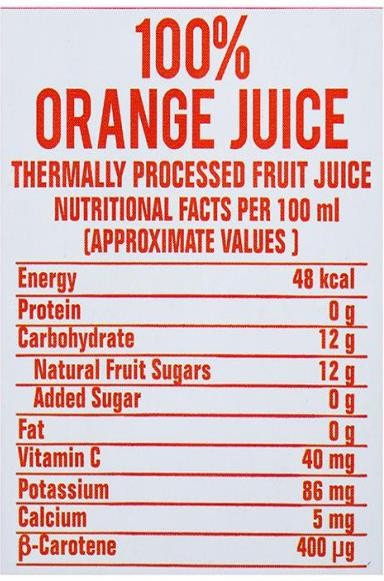


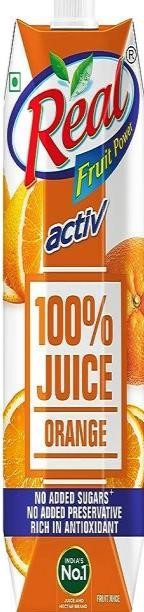

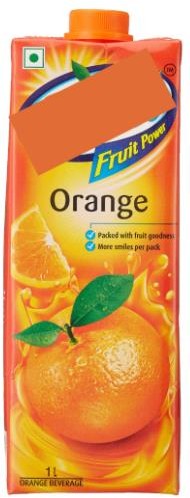

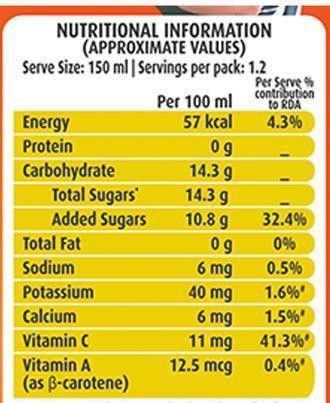


# FRUIT JUICE X FRUIT JUICE Y

| **S.no.** | **Statement** | **Fruit juice X (a)** | **Fruit juice Y (b)** | **Don’t know (c)** |
| --- | --- | --- | --- | --- |
| i.) | Which fruit juice has higher energy content? |  |  |  |
| ii.) | Which fruit juice is overall healthier? |  |  |  |

1. Virat Kohli features in an advertisement for a chocolate drink powder. He says that these powders increases stamina, enhances immunity, sharpens mental memory and is the reason for his energy!

1A) Do you believe that chocolate drink powders increase the immunity?

a. Agree b. Disagree c. Don’t know

2A) Do you believe that chocolate drink powders increase intelligence?

a. Agree b. Disagree c. Don’t know

3A) Do you believe that chocolate drink powders are a part of Virat Kohli’s daily diet?

a. Never b. Sometimes c. Always d. Don’t know

**QUESTIONS EXCLUDED FROM SCORING**

In general, how well do you understand the nutritional information provided though the following channels?

| **S.no.** | **Source** | **I understand (a)** | **I don’t understand (b)** | **I do not make use of this kind of information (c)** |
| --- | --- | --- | --- | --- |
| 1. | Food labels |  |  |  |
| 2. | Newspapers |  |  |  |
| 3. | TV or radio program |  |  |  |
| 4. | Nutritionist/ Dietitian |  |  |  |
| 5. | Medical doctor |  |  |  |

How frequently do you consume the following foods?

| **S.no.** | **Food groups** | **Daily**  **(a)** | **Thrice a week**  **(b)** | **Once a week (c)** | **Once in two weeks (d)** | **Monthly once**  **(e)** | **Rarely**  **(f)** | **Never**  **(g)** |
| --- | --- | --- | --- | --- | --- | --- | --- | --- |
| 1. | One fruit |  |  |  |  |  |  |  |
| 2. | A glass (200mL) full of milk |  |  |  |  |  |  |  |
| 3. | Green leafy vegetables |  |  |  |  |  |  |  |
| 4. | At least one vegetable |  |  |  |  |  |  |  |
| 5. | Pulses/dals |  |  |  |  |  |  |  |
| 6. | Egg |  |  |  |  |  |  |  |
| 7. | Chicken/ mutton/ fish |  |  |  |  |  |  |  |
| 8. | Pizza, pasta, burger |  |  |  |  |  |  |  |
| 9. | Noodles, manchurian, |  |  |  |  |  |  |  |
| 10. | Samosa, pakoda, vada |  |  |  |  |  |  |  |
| 11. | Panipuri, chaat |  |  |  |  |  |  |  |

Read the following statements and rank their options accordingly. 1 being the highest rank and 7 is the lowest rank.

| **S.no.** | **Food groups** | **Family**  **(a)** | **Friends**  **(b)** | **Internet (You Tube, website) (c)** | **Once in two weeks**  **(d)** | **Dietitian**  **(e)** | **Medical doctor**  **(f)** | **Books**  **(g)** |
| --- | --- | --- | --- | --- | --- | --- | --- | --- |
| 1. | In your daily living, from whom do you get the food and diet related information? |  |  |  |  |  |  |  |
| 2. | Whom do you trust the most to get the food and diet related information? |  |  |  |  |  |  |  |

Tick the following options based on your personal practice.

| **S.no.** | **Food groups** | **Never**  **(a)** | **Sometimes**  **(b)** | **Always**  **(c)** |
| --- | --- | --- | --- | --- |
| 1. | Do you change your eating habits based on dietary advice you have received in the media (newspapers, magazines, internet, TV)? |  |  |  |

**SCORING SHEET FOR THE TOOL**

| **Question number** | **Option** | **Scoring** |
| --- | --- | --- |
| 1-16, 18-24, 27, 30 | - Correct answer - Wrong | - 1 - 0 |
| 17.1-17.3, 17.5, 25.3 | - Agree - Disagree | - 1 - 0 |
| 17.4, 25.1, 25.2, 31.1A-31.2A | - Agree - Disagree | - 0 - 1 |
| 28.5-28.8, 31.3A, 26 | - Never or Sometimes - Always | - 1 - 0 |
| 28.1-28.4, 28.9, 29.1-29.12 | - Never or Sometimes - Always | - 0 - 1 |

*****Subjects ticking don’t know are directly allotted a score of zero for any question.* ***Total score is 73.***

**CUT OFF SCORES FOR THE LEVEL OF NUTRITION AND FOOD LITERACY**

| **Cut off scores** | **Interpretation** |
| --- | --- |
| ≤40 | Low level |
| 41-55 | Medium level |
| ≥56 | High level |
